# 254 What is the cost of family-based physical activity interventions? A scoping review

**DOI:** 10.1093/eurpub/ckae114.278

**Published:** 2024-09-26

**Authors:** S Maria O’Kane, Chloë Williamson, Marie H Murphy, Alison M Gallagher, Angela Carlin

**Affiliations:** Institute of Nursing and Health Research, Ulster University, Derry/Londonderry, BT48 7JL, UK; Physical Activity for Health Research Centre (PHARC), Institute for Sport, Physical Education and Health Sciences, University of Edinburgh, Edinburgh, EH8 9YL, UK; Physical Activity for Health Research Centre (PHARC), Institute for Sport, Physical Education and Health Sciences, University of Edinburgh, Edinburgh, EH8 9YL, UK; Centre for Exercise Medicine, Physical Activity and Health, Sports and Exercise Sciences Research Institute, Ulster University, Belfast, BT15 1ED, UK; Nutrition Innovation Centre for Food and Health (NICHE), Biomedical Sciences Research Institute, Ulster University, Coleraine, BT52 1SA, UK; Centre for Exercise Medicine, Physical Activity and Health, Sports and Exercise Sciences Research Institute, Ulster University, Belfast, BT15 1ED, UK

**Keywords:** Family, physical activity, interventions, costs, cost analysis

## Abstract

**Introduction:**

Globally, 81% of children fail to meet physical activity (PA) guidelines and the causes of such inactivity are complex and multifactorial. Parents can influence the PA levels of their children through modelling, co-participation, encouragement and family support. A meta-analysis by Brown *et al* (2016) showed family-based PA interventions to be effective. However, there are many perceived barriers to family PA and it is accepted that there is a persistent socioeconomic gap in PA. Socioeconomic status is an important determinant of health and can influence attitudes, experiences, behaviours, and exposure to health risk factors. PA is positively associated with socioeconomic status and recent research indicates that children from the least affluent families are less likely to meet PA guidelines in comparison to children from the most affluent families (44% vs 55% meeting PA guidelines respectively). To support families to address these inequalities and improve population health, an understanding of the costs associated with family-based PA interventions is required. Therefore, the aim of this scoping review is to provide a comprehensive overview of health economic evaluations of family-based PA interventions.

**Methods:**

This scoping review protocol will be registered with the Open Science Framework and will be conducted according to the Arksey and O’Malley framework for scoping reviews. Electronic databases (Ovid MEDLINE, Embase, Web of Science, PubMed, Cochrane Library, and Scopus) will be searched from inception. Reference lists of eligible studies and relevant systematic reviews will be searched for potentially eligible studies. Included studies will be those that recruited a family unit, delivered an intervention that included a component of PA (e.g. exercise, sport, play), examined the effect on PA and reported on the economic costs. The titles and abstracts of potentially relevant articles will be screened independently by two reviewers and data from eligible studies extracted using Covidence software. Data will be charted and the results summarised.

**Conclusions:**

This scoping review will provide an understanding of the costs associated with family-based PA interventions, information that will be important for the development of evidence-based interventions and for future policy and commissioning.

